
JOURNAL INFORMATION
==============================
NLM Title Abbreviation: Med Chir Rev
NLM Journal ID: 0034617

The Medico-Chirurgical Review

Publisher: Biomedical Journal Digitization Project

ARTICLE INFORMATION
==============================
PMCID: PMC4966260
PMID: 29917630
Article version: 1
Subjects: Reviews, Diseases Produced by Malaria

Researches into the Causes, Nature, and Treatment of the Diseases of India, and of Warm Climates Generally

Print publication date: 1828 Oct 1
Volume: 9
Issue: 18
First page: 337
Last page: 347
1828 Aug
License: This work is free of known copyright restrictions. For more information, please see PMC Back Issue Digitization.
License URL: https://creativecommons.org/publicdomain/mark/1.0/

==============================

### VII.

### *Researches into the Causes, Nature, and Treatment of the Diseases of India, and of Warm Climates generally.* By JAMES ANNESLEY, Esq. &c.

[ART. II.—ACUTE HEPATITIS.]

IN a former article, (No. XVI. p. 409, &c.) we presented our readers with an analysis of the first half of Mr. Annesley's first volume on Indian Diseases. We now resume our labours—we may well say *labours;* for it has never been our lot to see valuable matter so diluted, and, comparatively speaking, *lost,* in a deluge of superfluous verbiage, as in these volumes. We understand that the East India Company make the purchase of this work nearly imperative on their medical officers. If so, Mr. Annesley has imposed a *heavy* tax on his Oriental brethren. We do not allude to the FOURTEEN GUINEAS—though that is something to an assistant surgeon on first setting out—but, to the expense and inconvenience of employing a buffalo for the transportation of these volumes from station to station in the East Indies. That "a GREAT BOOK IS A GREAT EVIL," will be amply verified in this instance—and many a time will "MEGA BIBLION"! be heard, in uncouth dialects, "o'er lofty Ghaut, through lonely glen," as aspirated by the panting Coolie, while groaning beneath the weight of a—"GREY GOOSE QUILL"! Well might MIRABEAU exclaim, in the National Convention, "WORDS are THINGS." They are indeed—and very *heavy* things sometimes! Had Mr. Annesley been acquainted with the discovery of our friend LACON, and applied the VERBIFUGE to his proof-sheets, he would have *lightened* the labours of many a personage, besides the buffalo and Coolie! But it is now too late, and we can only endeavour to diffuse the materials of these costly and highly valuable volumes in a more *portable* form than the author has thought proper to adopt.

Hepatitis is a word which almost universally, in this country, calls up the idea of a hot climate, and especially the climate of India. There is hardly any acute inflammation less common in Europe than that of the liver—none so frequent as this phlogosis in our Asiatic dependencies. The reasons why hepatitis should prevail more generally in India than England have been discussed, but not finally adjusted. Some place the etiology of the disease to the account of atmospheric heat—some to a specific miasm, peculiar to India—and others to the luxurious living of Anglo-East Indians. It is probable that the same miasm which produces jungle, marsh, and the whole tribe of remittent and intermittent fevers, gives origin also, in many instances, to dysentery, cholera, and hepatitis.

Mr. Annesley observes that—"inflammation of the liver generally *supervenes,* either as a *primary* disease, without any very apparent state of previous disorder, or as a consequence of one or more of the functional derangements, &c." We notice this opening sentence of the section to shew how fond the author is of words, and how little he cares about their meaning. How can hepatitis

*supervene* as a *primary* disease? If it *supervenes* at all, it must supervene on some other disorder. Even the latter part of the sentence is clumsy, if not incorrect. If inflammation of the liver is a mere *consequence* of functional disorder, the word supervene is still an inappropriate one. We seldom indulge in verbal criticisms; but the work under review certainly requires much *verbal* expurgation.

The right lobe of the liver is more frequently inflamed than any other part—the left lobe less frequently. The parenchymatous structure, in Mr. A.'s experience, is much oftener the seat of phlogosis than the coverings of the liver. Frequently, indeed, we find the substance of the liver destroyed by inflammation or abscess, without any appearance of phlogosis on the surface.

"Inflammation of the substance of the liver seldom commences with a well-marked rigor or chill, unless after exposure to a powerful exciting cause operating upon the system from without, as cold or wet, currents of air, night dew, or malaria. When chills or rigors mark commencing inflammation of the internal structure of the organ, there are generally one or more of the symptoms we have enumerated as characterising congestion also present. Indeed, a congested state of the organ about to be diseased always accompanies that particular condition of system which gives rise to rigors, if it does not actually cause this particular phenomenon; and it generally accompanies inflammation of the substance of the organ, to a greater or less extent, throughout its progress. The patient usually complains, about this time, of oppression, weight, and uneasiness about the pit of the stomach and right hypochondrium, extending sometimes under the ensiform cartilage, and in the direction of the diaphragm and mediastinum to the back and shoulder blades. These symptoms are usually increased upon a full inspiration, taken at the time when pressure is made beneath the ribs, or when pressure upon the stomach and back is made at the same time. The pulse is, at this very early period of the disorder, scarcely affected; but it soon becomes accelerated towards night; it is often slower and more oppressed than usual, and occasionally irregular or remittent. The countenance is now usually pale, sallow, or somewhat anxious; the spirits considerably depressed; the tongue yellowish, white, and more or less foul, and the patient complains of loss of appetite and of sickness, with an unpleasant taste in his mouth. The bowels are often irregular, but at first generally costive, and the urine is in small quantity, loaded, and high coloured. There is sometimes headach, and generally a disturbed sleep, and often slight dyspnœa and sighing, with slight oppression at the chest and epigastrium.

"As the disease of the internal structure of the liver advances, the pulse becomes quicker, fuller, and more irritable in its beat during the evening and night, and it is often oppressed and embarrassed during the morning and day, and sometimes throughout, unless copious depletions have been practised early in the disorder; the sense of uneasiness in the region of the liver and epigastrium is often augmented; and if vascular fulness of the organ be great, and particularly when the inflammation results from congestion, the patient complains of a heavy, dragging pain, increased on sudden motion, or by turning suddenly in bed. There is often a short, suppressed cough, dyspnœa, with shortness of breathing, a catch in the respiration, particularly after quick motion. Upon examination, in these cases, tumidity of the viscus may be often ascertained from its protrusion beneath the ribs and scrobiculus cordis. The easiest position is usually upon the back, or sitting gently bent forward. All these symptoms are generally increased upon taking matters into the stomach; and the pulse is now much accelerated, especially towards evening. Difficulty of lying upon the right side is not frequently present, and pain in turning to the left side is not often felt, unless the change of position be made suddenly. The tongue at this stage of the disease is generally coated, and of a yellowish or brown colour; it is frequently also dry, particularly at its middle. The pain sometimes complained of at the top of the right shoulder, and so improperly stated as being one of the chief signs of hepatitis, is, when present, certainly characteristic of the disease in the right lobe; but, unfortunately, this symptom is only occasionally present; and the inexperienced practitioner, who has been taught to look to this as a distinctive mark of the disease, infers, when it is not observed, that the liver is sound. With respect to the pain actually accompanying inflammations of this organ, we may state that it is often felt in the region of the liver, in the lower part of the thorax, and in the epigastric region: it is sometimes referable to the top of the right shoulder, frequently to the right shoulder-blade, and occasionally to both scapulæ: it is, on some occasions, seated in the back, between the lower angles of the scapulæ, and, in some instances, the only pain which has been complained of has been in the loins. We have observed it, in a few cases, in the right clavicle and its vicinity; and in others, in the left shoulder and shoulder-blade only. In many cases, pain is increased in the situation of the disease, or its vicinity, upon quick motion, upon making a false step, or upon turning suddenly from one side to the other; and, in a few obscure cases, pain is complained of only on such occasions. When the internal structure of the organ is affected, the pain in the hypochondriac and epigastric regions is seldom acute; there is most frequently a sense of aching or dragging, with oppression at the præcordia. Pain is seldom acute, tensive, or pungent, unless the surfaces or ligaments become affected. There is usually great anxiety at the epigastrium and præcordia, accompanied with frequent sighing, particularly when pressure is made simultaneous on the right hypochondrium and under the right shoulder-blade. We have seen a few cases where pain followed the course of the muscles of the right side of the neck: it often extends from under the ensiform cartilage, in the direction of the mediastinum, to between the shoulder-blades; and when this is observed, oppression, dyspnœa, or a sudden catch in breathing, and a dry cough, generally accompany it. Pain frequently, also, extends from the right side, under the shoulder blade, to the spine, where it terminates. On many occasions, when great congestion of the vessels of the liver seems to accompany inflammation of its substance—states of the organ which, as we have already said, are frequently co-existent—the right lobe becomes very much enlarged, and rises up into the right cavity of the thorax, occasioning great oppression at the chest, fulness at the epigastric region, dyspnœa, frequent dry cough, and sometimes acute pain, owing to the great distension of the covering of the liver at this part, with an increased discharge of mucus from the bronchi. In such cases, the exacerbation of pain in the chest, upon a full respiration or on coughing, the flushed or tumid state of the countenance, occasioned by the interrupted circulation through the lungs and the seat of the complaint, are apt to make the inexperienced practitioner mistake the disease for pneumonia. In cases of this description there is generally more or less pain or uneasiness felt about the shoulder-blades, or top of the right shoulder, or between the scapulæ; and often numbness of the right arm, with pain about the insertion of the deltoid muscle, or at the wrist, is complained of; rarely, a slight numbness or pain is also felt down the right hip." 419.

Nausea and vomiting are often concomitants of the more acute attacks, and generally indicate that the inflammation is seated near, or is extending to the stomach, or in the direction of the ducts. This more usually happens when the inflammation results from accumulations of vitiated bile.

"In such cases the patient complains of sense of fluttering, weight, and fulness, at the right hypochondriac and epigastric regions,—sometimes of pain in the abdomen,—and he reclines chiefly on the left side; the stools are generally watery, frequent, scanty, and very dark coloured, with tenesmus and many of the symptoms of dysentery, for which disease it is often mistaken. Even when but little sickness at stomach is present, there is always loss of appetite in the more acute forms of the disease, thirst, heartburn or gripes about an hour or two after a meal, and considerable low spirits; and the patient often reclines upon the back or left side, in preference to any other position." 419.

As the inflammation advances, the fever, and especially the evening exacerbation, becomes more marked—the tongue is generally covered with a white or yellowish brown fur—moist in the beginning, but dry in the advanced stages.

In cases where the hepatitis has supervened on previous disorder of the alimentary canal, or after repeated attacks of hepatic disorder, "the tongue seems often smooth and glossy, marked by fissures, and lobulated." These are bad signs. The bowels are generally much disordered—the motions being vitiated, scanty, slimy, watery, or of a dirty brown colour. The thirst is urgent, and the nights are restless.

"The state of the countenance and skin deserves attention during the progress of disorder. At its invasion, particularly when attended with chills or rigors, the countenance is pale or sallow, and the skin shrunk and pale on the extremities, but often natural in the trunk. As the inflammatory action becomes developed, the countenance fills out more fully; and when there is great fulness and oppression in the region of the liver and chest, the face often becomes fuller than natural, with some degree of dusky redness in the cheeks. The countenance and eyes, however, still possess a murky, or muddy, or sallow hue, and more or less of a dark circle surrounds the eye, particularly beneath it. The tunica albuginea is either of a yellow tint, or of a dull white or pearly hue. The patient often complains of pain in the forehead and over the eyes. The skin on the trunk, especially towards evening, is generally warmer than natural, and is sometimes attended with a greasy feel, and a scanty or partial perspiration. When perspiration is copious, it is frequently very offensive. A certain degree of jaundice is often remarked in the hepatitis of Europe, especially when it terminates in abscess; but jaundice is not a frequent concomitant of hepatitis in India, unless when the ducts or gall-bladder become involved in the disease, or when it supervenes to biliary calculi and obstruction of the ducts. The countenance and eye are, however, always deficient of clearness, and possess a sickly expression." 421.

The urine is high-coloured, scanty, loaded, and produces a sense of scalding when passed. A dysenteric state of the bowels is a common concomitant—and healthy bile is hardly ever found in the stools. Mr. A. observes, what no practical man will doubt, that there is no *one* symptom or phenomenon on which we can depend, as pathognomonic of active inflammation in the liver—and he might have added, of inflammation in any other internal structure.

When the surface of the liver becomes inflamed, whether primarily or secondarily, the symptoms assume a more acute and definite character.

"Febrile signs are more prominent, and often supervene to slight rigors and chills; the pulse is generally much accelerated, and hard; the pain in the right hypochondrium is more or less acute; and when the upper surface of the right lobe is affected, or when great tumefaction of this part is present, so that it rises up into the chest, considerable pain and tension are also felt in the right thorax and under the ensiform cartilage and sternum, so as to resemble an attack of pleuritis. There is also cough, much increase of pain, or a catch, upon a full inspiration, or upon pressure, especially when made at the time of a full inspiration. When the whole of the upper surface of the organ is the seat of inflammatory action, the attack may be mistaken for pneumonia. The oppression, difficulty of breathing, pain in the course of the diaphragm and under the sternum, being generally considerable." 424.

The heat and dryness of skin and tongue are also greater in the membranous than in the parenchymatous inflammation. The secretions from the bowels are very variable in this form—generally they are diminished in quantity, and sometimes deficient of bile. Diaphragmitis is not unfrequently superinduced in this form of hepatitis, from extension of the inflammation—and the lungs also are not uncommonly inflamed, attended with great tension in the hypochondria and inconvenience in breathing. The cough is hard, frequent, and suppressed as much as possible by the patient. When the outer surface of the right lobe is inflamed, the patient lies best on that side, and has pain extending round to the right scapula—sometimes to the shoulder. On the other hand, when the concave surface of the organ is the seat of inflammation, the functions of the stomach are prominently disturbed.

"Nausea and vomiting are often present, particularly a few minutes after substances are taken into the stomach. The thirst, anxiety, and pain at the epigastric region, are urgent, and there is usually much pain in the back, and sometimes in the right shoulder and muscles of the right side of the neck. The pulse is variable, but generally irritable, quick, small, contracted, or hard. There is often felt a sense of fluttering at the scrobiculus cordis, with a heavy dragging pain in the same situation; anxiety and frequent sighing; and sometimes, in the advanced state of the disease, hiccup is present, especially after cold fluids are taken into the stomach. The patient generally reclines upon the left side, or leans gently forwards. All these symptoms become more urgent if the inflammatory action have extended to the gall-bladder, to the ducts, or to the stomach itself. When such is the case, there is generally a sense of burning felt at the epigastrium, with fulness, frequent and painful eructations of flatus, very quick pulse, with cold, clammy hands, and increased heat of the trunk. The vomiting is frequent and painful, the urine in small quantity, and the stools watery, scanty, and often morbid and offensive. When the ducts and gall-bladder are affected, the pain is felt darting to the right side and back, and from under the ensiform cartilage, in the course of the mediastinum, to the spine; sometimes it extends from the epigastrium to the umbilicus, and back to the right hypochondrium. Singultus and acrid eructations not infrequently also supervene as the disease advances, particularly after substances are taken into the stomach. The patient can seldom bear pressure on the right side and epigastric region, and feels increased uneasiness upon a full inspiration. Increase of uneasiness merely cannot, however, be considered as a distinctive sign of the seat of the inflammation, as this function is more or less affected, particularly on attempts to fill the lungs, in all the stages and forms of the disease: the degree, however, to which the breathing is affected, and the seat of pain or uneasiness, upon taking a full inspiration, is often a guide to the actual state of disorder. A similar remark may be applied to the pain and uneasiness frequently felt upon making a forced expiration: for this means of ascertaining the seat of pain ought always to be practised, whenever the exact nature of the case is in any way doubtful. There are also observed great restlessness and want of sleep, a foul state of the tongue, with large, foul, and brown papillæ. If the tongue become clean from the treatment, the papillæ generally remain long excited or prominent." 427.

It is but seldom that the left lobe of the liver is alone inflamed—and indeed it may be said that membranous inflammation is seldom unaccompanied by an extension of the phlogosis, more or less, into the substance of the organ. The symptoms will then, of course, partake of the characters peculiar to both forms.

### Pathological Appearances.

Excepting where people die of other diseases, as fever or dysentery, it is seldom that the pathologist has an opportunity of observing the appearances presented by the early stages of hepatitis. When such opportunities do occur, he often finds some part of the surface or interior of the organ evincing the usual signs of phlogosis—vascularity, redness, gelatinous coatings on the *surface*—increased vascularity, redness, and friability or softness in the recently inflamed *parenchyma*.

"In some instances, the surface of the inflamed organ is variously shaded. Sometimes, it is marked with red, brown, brick-coloured, greenish-brown, and even with almost black spots and streaks, while the internal structure is inflamed, congested with blood, much tumefied, and softer than natural. Upon making a section of the viscus with a very sharp scalpel, and after wiping with a sponge the cut surfaces, they present a lighter-coloured reticulum, or mesh, studded with red or brick-red granulæ, and the divided ends of blood-vessels and biliary ducts. Upon being torn asunder—which is generally done with more facility in the acutely inflamed state, although sometimes with more difficulty in the chronic conditions of disease—the torn surfaces exude a greater quantity of fluid blood, but still retain their minutely granulated structure, and present both a brighter and a deeper colour than in their healthy state. When abscess forms in the substance of the organ, then the appearances become very materially and very variously altered." 434.

Gangrene has been remarked by many writers and teacheis—but our author, whose opportunities have not been exceeded by any other practitioner, never saw a case of the kind. It is probable that the black congested and softened state above-described, has been mistaken for gangrene. The inflammation and its consequences frequently spread, of course, to the neighbouring parts, and the stomach, duodenum, colon, lungs, and even the kidneys, are often involved in the destructive process. Sometimes the liver is found tumid and congested—the ducts either choaked with inspissated bile, or reduced to an impervious cord. This constriction of the duct appears, in some cases, at least, to be the result of spasm—in others, of organic change from preceding inflammation. Our author very properly enjoins a careful manual and occular examination of the region of the liver in all cases.

"Manual examination should, therefore, be resorted to on every occasion; and the trunk of the body should, in all cases, be exposed to the view of the practitioner, in order to ascertain if fulness or bulging exist in any part of the hypochondrium or in its vicinity. When manual examination is being made, one hand of the practitioner should be pressed at first gently upon the part between the base of the right shoulder-blade and the spine, whilst with the other he endeavours to detect, gently, delicately, and with refined tact, tenderness, fulness, or distension, either beneath the right false ribs, at the epigastric region, to the left of this region, or between the right hypochondrium and umbilicus. The state of the intercostal spaces should also be examined on the right side; and if pain be complained of in any of these situations, its nature may be inquired into by careful and varied pressure, whilst counter-pressure is being made on the back, in the place pointed out. The patient ought also to be made to breathe fully at the time when this examination is going forward, and he may be directed to bend, or move his body in various directions. If fulness, tumefaction, or distinct tumour, be felt, the practitioner should endeavour to ascertain their nature by gentle and varied pressure with the points of the fingers; and the existence of tenderness, the degree of tenderness, the depth at which it seems to be seated, and the presence of fluctuation, whether obscure or palpable, ought to be inquired into with as much dexterity as the practitioner can command." 437.

### ETIOLOGY.

Amongst the first causes of hepatic inflammation, Mr. A. places all those which "disorder the functions of the stomach, and at the same time derange the circulation in the biliary organs." This vagueness of expression is not much cleared by what immediately follows:—"These are whatever directly or indirectly produces a plethoric state either of the vascular system generally, or of the digestive organs, with debility." In a subsequent page we have a more tangible catalogue of the causes which produce hepatitis in our Eastern possessions. These are, full living, especially on animal food—high-seasoned dishes—high temperature—moisture—malaria—neglect of the bowels—indolence—puberty—wine—insolation—depressing passions, and all the various causes of dyspepsia.

It is curious that the age of puberty seems to be so operative in disposing to hepatitis. The disease is seldom or never met with amongst Europeans before that epoch. There are many circumstances, however, which call into play the various causes of gastric and hepatic affections after the age of puberty, which were previously inoperative. Among the causes enumerated, Mr. Annesley dwells strongly on the effects of bad water. The depressing passions are consequences and causes equally of the disease in question. The influence of atmospheric heat in the production of hepatic derangements is now acknowledged almost universally; and it is on this principle chiefly, that we can account for the comparative frequency or infrequency of the disease in different parallels and localities of our Indian empire.

"A varied observation in different provinces in India has furnished us numerous proofs in illustration of the very extended influence of this cause amongst the natives of temperate climates; and we need only refer to the Abstracts of the Returns (given at p. 110 *et seq.*) and in the Appendix to the present Volume, for proofs of this influence on an extended scale. In the Carnatic, the prevalence of hepatic disease is well known: here the range of temperature is much higher than in any other part of India; the fall of rain is also very much less than in the provinces under the Bengal and Bombay presidencies; and the soil more naked, more gravelly, and less retentive, than in the latter: hence the great heat is not so frequently nor so adequately abated; and the cooling effects of a fall of rain sooner cease. Whilst the provinces under the Madras presidency are near the equator, several of the other districts of the British empire in India are situate beyond the tropic; and thus, from latitude, and the various peculiarities of soil, situation, and climate, the Carnatic and several other provinces in the Indian peninsula possess a much higher range of temperature, and a proportionately greater liability to inflammatory affections of the liver. It is sufficient for us to express the result of our own observation respecting the matter, since the same fact has been very justly stated and illustrated in Dr. James Johnson's valuable work on tropical diseases, which is deservedly in the hands of every practitioner in warm climates." 441.

A series of very instructive cases of acute inflammation of the liver are here introduced by Mr. Annesley, some of which we shall abridge in the Periscope of this number. One case will be sufficient to illustrate the treatment of acute hepatitis, to which we shall next proceed.

"*Case.* Richard Kelly, (had been several years in India,) was admitted the 15th March, 1817, with the symptoms of congestion of the liver and accumulation of bile. These were removed by an emetic and purges. Five days after his discharge he returned, and on the evening of the 22d, the following report of him was given in the hospital journals:—Attacked with severe aching pains in the loins, shoulder-blades, and right side; anxiety; cold partial sweats; foul and excited tongue; and quick pulse.—Apply twenty leeches to his side. Calomel, gr. xx. h. s. s.

23d.—The pain in his back and side is relieved; but he has still occasional pain under the ribs: he has also some pain in his right shoulder. Tongue white and excited; pulse 80, small, irregular, and irritable. The pain is increased on a full inspiration. Was purged in the night.—Twenty-four leeches to the side; and the mist. purgans, with half an ounce of sulphate of magnesia.—*Evening.* Pain of the side nearly gone, but he complains of soreness over his whole body; pulse 96; tongue dry, furred, and excited; great thirst; stools crude and copious.—Calomel. gr. xx. h. s. Mist. salina febr. A blister to his side.

"24th.—Stools crude and full of viscid mucus; pain in his side and shoulder gone; tongue white and furred; pulse 78.—Pulv. purgan. ʒjss. stat. Rub in ʒj. unguent. mercur. thrice daily. Pilul. hydr. cum calom. no. 1 ter die. Haustus amar. cum sennâ, ℥ij. primo mane.

"This treatment was continued during the 25th and 26th. The pulse became less frequent; his tongue cleaner; and his motions more natural. On the 27th, he had a slight return of pain in the night, beneath the fifth rib of the right side; but his tongue and stools were natural at the morning visit; his pulse 66; and his skin cool.—Twelve leeches were applied, and the pilul. hydr. cum cal.; the mercurial friction and haust. amar. cum sennâ were continued.—*Evening*. Can breathe with perfect ease; no complaint.—On the 31st, the mercurial friction was diminished; the pilul. hydr. cum. cal. was omitted; and five grains of blue-pill given every night; the saline mixture through the day; and a dose of the purging mixture early in the morning.

"On the 2d April, he was perfectly well; his bowels, tongue, skin, and pulse, being perfectly natural. The blue-pill and mercurial friction were left off; and a dose of the purging mixture given occasionally. He was discharged on the 4th.

"*Remarks.* This case illustrates chiefly the connexion often existing between functional disorder and inflammation of the organ. The symptoms were clearly referrible, in this case, to the liver, and indicated disease affecting principally its internal structure. In this case there was no rigor at its commencement; and the pain of the shoulder was at first not present. General soreness, after the pain had been removed by depletions, was here complained of, and is a frequent symptom of inflammation of the internal structure of this important organ." 448.

### TREATMENT OF HEPATIC INFLAMMATION.

This is one of the most important subjects which a tropical writer can descant on—especially as there is some discrepancy of opinion, not so much in respect to the nature of the remedies, as to the extent of their application.

*General and local bleeding.* Although this is the most powerful of all therapeutical agents, even between the tropics, yet it is not *there* practised to the extent which Mr. A. deems proper. This, of course, is owing to a lingering portion of ancient prejudice. The following are his sentiments on this head.

"We can truly say, as respects the different forms of inflammation of the liver as they occur in India, that we have had occasionally to regret not having practised blood-letting when it might have been attempted with hopes of success, or carried it sufficiently far to be really beneficial; but we have never found that mischief resulted either from its performance, or the extent to which it had been pushed. Numerous instances, on the other hand, have come before us where,—from early education, the indulgence of prejudices, an indifference to the examination of those who have died of this class of diseases, and, consequently, from an insufficient acquaintance with the nature and extent of disease which occasioned death,—sufficient vascular depletion had been neglected at that stage of the malady when it might have been most serviceable, and the formidable consequence of disease prevented." 583.

Leeches in India are abundant and of excellent quality—consequently local depletion, to any amount, may be expeditiously effected. In the majority of cases, the local is preferable to the general bleeding—though the latter is indispensable amongst those who are fresh from Europe, full-blooded, and robust. Mr. Annesley ascertained that Indian leeches, on an average, extracted an ounce and a quarter each, besides what flows from the bites. In the active forms of hepatitis then, and among recently arrived Europeans, Mr. A. took one or two bleedings from the arm—the first carried to the point of making a sensible impression on the vascular system. When the excitement returned, from 16 to 30 leeches were applied to the right hypochondriac and epigastric regions. If the symptoms were not considerably relieved by these general and local depletions, the leeches were re-applied, and a large hot poultice laid over the bites, these last being stopped from bleeding previously. Mr. A. attaches great importance to the poulticing practice, especially where the hepatic disease is complicated with biliary derangement and dysenteric symptoms. The repetition of the local bleedings must, of course, depend on circumstances that require no detail here.

Among those who have resided long in India, local depletion will be generally sufficient, even in the most acute forms of the disease. With soldiers who have been addicted to strong liquors during their tropical services, depletion must be very cautiously employed.

*Mercurials—purgatives, &c.* When we consider how much the function of the liver is deranged before and during the attack of hepatitis—and how morbidly altered are its secretions, we cannot be surprised that purgation, and especially mercurial purgation, should be very generally necessary.

"For this purpose, we have generally prescribed, immediately after the first vascular depletion, a full dose of calomel, as this medicine appears to us the most beneficial in inflammatory states of the system, the most active in eliciting a healthy secretion of bile, and the most efficient in dissolving that viscid and tenacious secretion which covers the mucous coat of the intestinal canal at the commencement of nearly all the disorders affecting the organs of digestion. Unless the patient has come under treatment early in the day, when we have given this medicine immediately, we prefer the exhibition of it at bed-time, as it will then not disturb the rest of the patient by its operation, and will have had time to produce its effects upon the secretions and secreting viscera before morning; when a brisk purgative should be given, in order to carry out of the system accumulated fæces, and those morbid secretions which the previous exhibition of the calomel had prepared for removal." 593.

Mr. A. prefers the compound powder of jalap to other purgatives, for the above purpose—next to it, castor oil, and lastly, the "black draught." It is of great consequence in this complaint that the patient should not be disturbed in the night—and, therefore, when the bowels are irritable, Mr. A. recommends an opiate—even when it is necessary to give calomel every night. The following observations are important.

"If the exhibition of twenty grains of calomel at bed-time, and a purgative in the morning, saline diaphoretics being given through the day, affect the mouth, which frequently happens when vascular depletion has been carried sufficiently far, ptyalism should be quickly induced; but after its supervention, mercurials ought to be laid aside for a time. The reason of our recommending the speedy induction of ptyalism after the mouth becomes affected, is an idea which we entertain respecting the influence of the constitutional effects of mercury upon inflammations of the liver; namely, that to induce the mercurial excitement of the vascular system, indicated by slight soreness of the gums, and to exhibit mercury or calomel in small quantities, frequently repeated with this view, is to keep up a state of slow inflammatory action in the secreting substance of the liver, which may of itself terminate in abscess; whilst, if the full operation of mercurial remedies be speedily induced, and ptyalism become abundant, a derivation from the seat of disease is occasioned to the mouth and salivary apparatus, the disease in the liver speedily subsides, and the functions of the organ are restored to their healthy state. We believe that much evil very frequently results from the general habit of giving too frequent doses of calomel, with a view of inducing the constitutional effects of mercury. Those who prescribe five grains of calomel every three or four hours, with this view, produce much greater irritation of the alimentary canal, are longer in obtaining their object, and exhibit much more calomel for the removal of the disease, than those who give twenty grains only at bed-time. This latter dose acts as a sedative to the irritable stomach in this disease, whilst smaller doses increase the irritability of this viscus when it is present, and often induce it where it was previously absent." 594.

If bowel-complaint exist or supervene, one or two grains of opium are to be combined with the calomel, and emollient enemata should be thrown up. In this way the calomel will soon affect the system, especially if vascular depletion have been sufficiently employed previously. Mr. A. is not an advocate for going on to the production of full ptyalism in those cases where the secretions become healthy before that event takes place, and the other symptoms subside.

"But if the secretions and stools still remain morbid; if any disorder can be detected, by a careful examination of the patient, in the seat of the liver or in the abdomen; if the tongue be not natural; and if the countenance be sallow or unhealthy,—the speedy induction of ptyalism will then often prove of service. If, however, we fail in inducing this effect in the course of four or five days, we shall generally find it detrimental to continue this plan any longer. The means by which the speedy induction of the mercurial action may be accomplished are various; but we have generally relied most upon mercurial inunction, performed thrice a day, with a combination of camphor with the mercurial ointment, the patient taking the usual full dose of calomel at bed-time, combined with James's powder, or antimonial powder and opium." 596.

Ptyalism once fully established, the mercurials are to be discontinued, and gentle tonics combined with alkaline carbonates are to be administered, together with saline aperients.

In the subacute and less active forms of Indian hepatitis, the use of saline purgatives, alternated with mercurials and alteratives, will generally be sufficient, after moderate depletion. But these failing, "the practitioner should endeavour, in the manner stated above, to induce, as speedily as possible, the full effects of mercury."

*Auxiliaries in the Treatment.* "There are very few remedies which are more deserving notice than the nitro-muriatic acid wash, and the internal use of nitric acid, in cases of acute hepatitis, after active depletions and mercury have been used: they promote the return of strength and the healthy establishment of the biliary secretion; and if deobstruent laxatives, with suitable regimen, be prescribed, and adhered to during their use, they remove obstructions, and promote a free circulation in the vessels of the liver. As a restorative of the energies of the system after mercurial courses, they have generally proved beneficial in our practice, particularly when conjoined with the cautious exhibition of gentle tonics, with light but nutritious diet, and suitable regimen." 600.

On the complications of acute hepatitis with pleuritis, gastritis, and inflammations of different contiguous organs, it is quite unnecessary to dwell, since the principles of treatment cannot be misunderstood, after what has been already detailed. From this article, the English reader will be able to form a very correct idea of the practice of medical men in the acute and dangerous inflammations of the liver between the tropics. Mr. Annesley is a man of such ample experience, sound judgment, and scrupulous fidelity, that every thing falling from his pen is highly valuable. If we have regretted the costly manner in which he has cast his volumes, and the unnecessary minuteness with which he has treated most of his subjects, we can assure him that not one of his most ardent admirers entertains more unfeigned respect for his talents and industry than ourselves. We cannot help again suggesting to him the propriety of publishing the letter-press of these volumes in a more condensed and less expensive form, leaving the plates to be purchased by those who can afford the expense.

In our next article we shall take up the important subject of Chronic Hepatitis, a disease now imported annually into this country, so as to make it a matter of anxious investigation with every medical practitioner. An accurate acquaintance with the acute forms of Indian Hepatitis, however, is indispensable to the study of the chronic forms, and for this purpose we have laid before the English reader a very comprehensive analysis of the facts and observations recorded in the great work before us.

---

### VIII.

*The Morbid Anatomy of the Bowels, Liver, and Stomach, Illustrated by a series of Plates, from Drawings after Nature, with explanatory Letter-press, and a Summary of the Symptoms of the Acute and Chronic Affections of the above-named Organs.* By John Armstrong, M.D., Lecturer, &c. Fasciculus I. and II. 4to. pp. 70. with ten coloured plates. Baldwin. June & July, 1828.

[Art. I. Fasciculus 1.]

Dr. Armstrong's works on Typhus, &c. which appeared some ten or twelve years ago, procured for their author a reception and patronage from the profession and general public, such as have never before been witnessed or experienced in the annals of medicine. From being a successful author, Dr. A. became, in a very short time, one of the most successful candidates for private practice, in this great metropolis, whither he came a total stranger—and, from a successful career in practice, he turned his attention to medical tuition, where he quickly drew a larger class of students than any other physician in London. Some people may say this was all good fortune—or chance. But we have an idea, that to take such a lead in three different and difficult walks of the profession—writing, practising, and teaching—requires something more than the chapter of accidents, the patronage of friends, or the caprice of fashion.* A single glance over the two fasciculi before us convinces us, had we no other rea-

---

* Whenever a medical man arrives at any distinction by his writings or his practice, three fourths of his *contiguous* brethren divide into two sects—one, (a very small party comparatively) extolling him to the skies, and seeing in him nothing but excellence—the other (a tolerably numerous band) depreciating the fortunate candidate, running down every thing that comes from his tongue or pen, and ascribing all his success to the indiscrimination of mankind, and the blindness of Fortune. Happily there is a third and very influential personage, called the public, who, being far removed from the sphere of private and personal feelings, forms a tolerably correct estimate of men and things in the long run. It was but the other day we heard the following fragments of a dialogue between two medical gentlemen who met in Regent Street:—

A. "Have you seen Dr. Armstrong's Plates?"

B. "Yes."

A. "Were there ever such *daubs* offered to the medical profession? The two fasciculi are a disgrace to any physician!"

Now if we might be allowed to judge, we would say, that the plates are very superior, but that we cannot vouch quite so much for the letter-press; though the meagreness of the latter may be accounted for by the circumstance that Dr. A. is preparing a larger work on the same subjects.
